# Cell cycle specific, differentially tagged ribosomal proteins to measure phase specific transcriptomes from asynchronously cycling cells

**DOI:** 10.1038/s41598-024-52085-5

**Published:** 2024-01-18

**Authors:** Jesse D. Cochran, Tess A. Leathers, Emir Maldosevic, Klara W. Siejda, Julian Vitello, Haesol Lee, Leigh A. Bradley, Alex Young, Ahmad Jomaa, Matthew J. Wolf

**Affiliations:** 1 Medical Scientist Training Program, University of Virginia, Charlottesville, VA USA; 2 Department of Anatomy, Physiology, and Cell Biology, University of California, Davis, USA; 3 Department of Molecular Physiology and Biological Physics, University of Virginia, Charlottesville, USA; 4 Department of Medicine, University of Virginia, Charlottesville, VA USA; 5 Department of Biomedical Engineering, University of Virginia, Charlottesville, VA USA; 6 Robert M. Berne Cardiovascular Research Center, University of Virginia, Charlottesville, VA USA; 7 Division of Cardiology, University of Virginia, Medical Research Building 5 (MR5), Room G213, 415 Lane Road, Charlottesville, VA 22908 USA

**Keywords:** Expression systems, Genomics, Mitosis

## Abstract

Asynchronously cycling cells pose a challenge to the accurate characterization of phase-specific gene expression. Current strategies, including RNAseq, survey the steady state gene expression across the cell cycle and are inherently limited by their inability to resolve dynamic gene regulatory networks. Single cell RNAseq (scRNAseq) can identify different cell cycle transcriptomes if enough cycling cells are present, however some cells are not amenable to scRNAseq. Therefore, we merged two powerful strategies, the CDT1 and GMNN degrons used in Fluorescent Ubiquitination-based Cell Cycle Indicator (FUCCI) cell cycle sensors and the ribosomal protein epitope tagging used in RiboTrap/Tag technologies to isolate cell cycle phase-specific mRNA for sequencing. The resulting cell cycle dependent, tagged ribosomal proteins (ccTaggedRP) were differentially expressed during the cell cycle, had similar subcellular locations as endogenous ribosomal proteins, incorporated into ribosomes and polysomes, and facilitated the recovery of cell cycle phase-specific RNA for sequencing. ccTaggedRP has broad applications to investigate phase-specific gene expression in complex cell populations.

## Introduction

Dynamic changes in gene expression influence the ability of cells to maintain identity, adapt to environmental conditions during homeostasis, respond to ligand-mediated signals, and coordinate growth. Organs and tissues are composed of heterogeneous populations of cells that can obfuscate the interpretation of gene expression profiles of specific cell subpopulations. Strategies to profile cell-specific gene expression include the physical isolation of cells of interest by laser capture microdissection or dissociation of single cells followed by cell sorting or antibody-based enrichment (immunopanning)^1^. Other strategies, including RiboTrap or RiboTag, employ the expression of transgenic constructs encoding epitope-tagged ribosomal proteins (for example, RPL10a or RPL22) to immunoprecipitate mRNAs from complex tissues^2–4^. TU-tagging uses the spatially restricted uracil phosphoribosyltransferase (UPRT) expression with 4-thiouracil (4TU) delivery to label and purify cell type specific-RNA from intact complex tissues^5–7^. Despite the advantage of purifying RNAs from specific cell types within complex tissues, these strategies track steady state gene expression across the cell cycle and are thus inherently limited by their inability to identify changes specific to cell phases.

More recent advances in single cell RNAseq (scRNAseq) have facilitated the molecular characterization of diverse cell types within tissues, organs, and whole animals^8–15^. There are, however, limitations to scRNAseq. scRNAseq methods require the physical dissociation of cells, a process known to alter the transcriptional landscape during the isolation process. These technologies also tend to survey the steady state gene expression across the cell cycle, limiting the ability to identify gene regulatory networks specific to G_1_, S, and G_2_/M in vivo. Moreover, scRNAseq can pose unique problems when attempting to isolate cell cycle-specific transcripts. Firstly, some cell types are not amenable to single cell sorting (i.e., cardiomyocytes) and subsequent single cell RNA sequencing^16^. Secondly, subpopulations can be too rare to adequately discern from artifact. Thirdly, low sequencing depth can limit the ability to accurately characterize cell subpopulations^17,18^. Finally, scRNAseq is significantly more expensive than bulk RNA sequencing. Alternative strategies are necessary to address some of these limitations.

Degron-mediated degradation is one mechanism used to regulate the expression of proteins during phases of the cell cycle^19–23^. FUCCI technologies employ chimeras of fluorescent proteins and degrons to detect cell cycling in vitro and in vivo^22,23^. In general, FUCCI reporters rely on the expression of SCF^skp2^ and APC^Cdh1^ to regulate Chromatin Licensing and DNA Replication Factor 1 (CDT1) and Geminin (GMNN) degrons. Second generation bicistronic FUCCI constructs contain internal protein cleavage sites, such as T2A from *Thosea asigna virus 2A,* to initially produce equimolar amounts of reporter proteins, whose subsequent levels are a function of the cell cycle phase via degron-mediated proteolysis^22^.

In this study, we merged two powerful strategies, the CDT1 and GMNN degrons of FUCCI and the epitope-tagged ribosomal proteins of RiboTrap/Tag technologies, to create cell cycle-dependent, tagged ribosomes named ccTaggedRP with the goal of discerning cell cycle phase-specific gene expression changes. The ccTaggedRPs were incorporated into ribosomes and polysomes as determined by sucrose gradient centrifugation, had similar subcellular location as endogenous RPL10a by immunofluorescence, and demonstrated predicted changes in protein expression that were dependent on the phase of the cell cycle. Here, we describe our first generation ccTaggedRP and employed the strategy to identify transcripts enriched during G_2_-M phase from asynchronously cycling HEK293-T cells. The findings also corresponded to transcripts identified by scRNAseq datasets of HEK293-T cells, suggesting that ccTaggedRP is a potential new strategy to detect differentially expressed transcripts during the cell cycle.

## Results:

### ccTaggedRPs are differentially expressed during the cell cycle and have similar subcellular locations as endogenous ribosomal proteins after transient transfection

**Figure 1:**
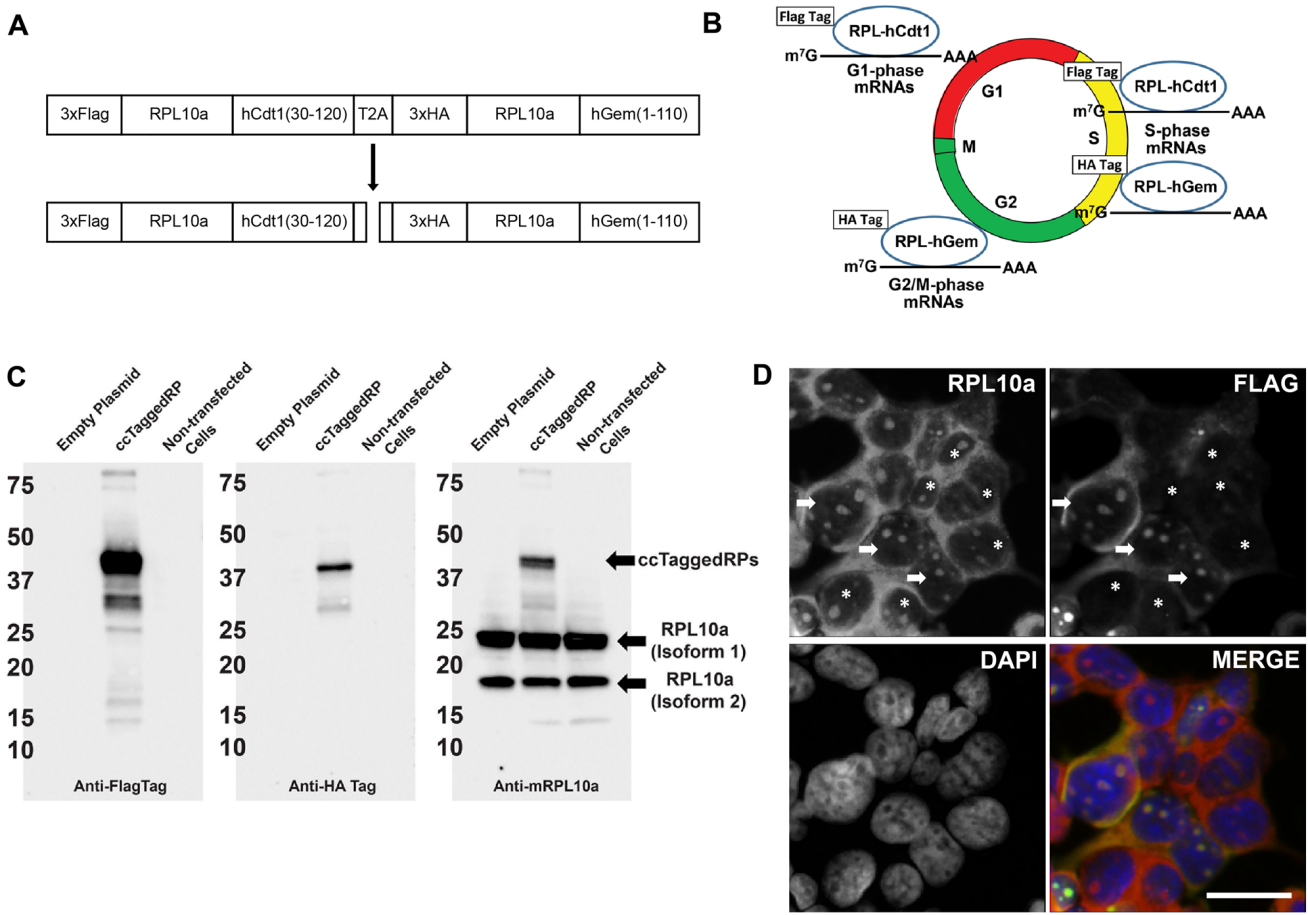
ccTaggedRPs are differentially expressed during the cell cycle and have similar subcellular locations as endogenous ribosomal proteins. **(A)** Schematic of the CAG-3xFlagTag-RPL10a-hCdt(30–120)-T2A-3xHA-Tag-RPL10a-hGeminin(1–110) construct and cleavage at the T2A site. **(B)** Schematic of the predicted change in 3xFlagTag-RPL10a-hCdt(30–120) and 3xHA-Tag-RPL10a-hGeminin(1–110) during the cell cycle. **(C)** Immunoblots of asynchronously cycling HEK293-T cells transiently transfected with empty plasmid, ccTaggedRP, or not transfected. Blots were probed with antibodies against Flag Tag, HA Tag, or mRPL10a. Bands corresponding to Flag recombinant protein, HA recombinant protein, and endogenous RPL10a isoforms are denoted by arrows. See Supplemental Fig. 6 for uncut blots. **(D)** Immunofluorescence of asynchronously cycling HEK293-T cells transiently transfected with ccTaggedRP stained for RPL10a and Flag Tag. Cells expressing ccTaggedRP are denoted by arrows. Cells not expressing ccTaggedRP are denoted by asterisks. DAPI was used to stain for nuclear DNA. DAPI is blue, RPL10a is red, and FLAG is green**.** Scale bars are included in the lower right corner and correspond to 10 μm.

To test the hypothesis that chimeras of epitope-tagged ribosomal proteins containing degrons would add new functionality to existing RiboTrap/Tag technology, we created a bicistronic 3xFlagTag-RPL10a-hCdt(30–120)-T2A-3xHA-Tag-RPL10a-hGeminin(1–110) (denoted “ccTaggedRP” hereafter). The encoded ccTaggedRP is translated as a single polypeptide and undergoes cleavage at the T2A site to initially produce 3xFlagTag-RPL10a-hCdt(30–120) and 3xHA-Tag-RPL10a-hGeminin(1–110) in equimolar amounts (Fig. 1A). However, the 3′ hCdt insertion targets the 3xFlagTag-RPL10a-hCdt(30–120) recombinant protein (denoted “Flag-RPL10a-hCdt” hereafter) for degradation during late S through G_2_ and M phase while the 3’ hGeminin insertion targets the 3xHA-Tag-RPL10a-hGeminin recombinant protein (denoted “HA-RPLA10a-hGem” hereafter) for degradation during late G_1_ through early S phase. Consequently, the steady-state concentrations of HA-RPL10a-hGem and Flag-RPL10a-hCdt recombinant proteins are dependent on the cell cycle phase. Specifically, Flag-RPL10a-hCdt and HA-RPL10a-hGem recombinant proteins are expected to be enriched in G_0_/G_1_ and G_2_-M, respectively (Fig. 1B). Of note, unlike FUCCI, the ccTaggedRP constructs do not contain fluorescent reporter proteins. The Flag-RPL10a-hCdt and HA-RPL10a-hGem recombinant proteins were expressed at the predicted sizes after transient transfection of HEK293-T cells with recombinant ccTaggedRP plasmid (Fig. 1C). Protein bands of lower molecular weight were observed in the Flag and HA blots, consistent with degradation products mediated by the hCdt(30–120) and hGeminin(1–110) degrons, respectively. Immunofluorescent staining of HEK-293 cells transiently transfected with ccTaggedRP showed similar patterns of Flag and RPL10a locations. Suggesting that the subcellular location of ccTaggedRP was similar to endogenous RPL10a in HEK293-T cells (Fig. 1D).

### ccTaggedRPs stably expressed in HEK293-T cells were incorporated into endogenous ribosomes and polysomes

**Figure 2:**
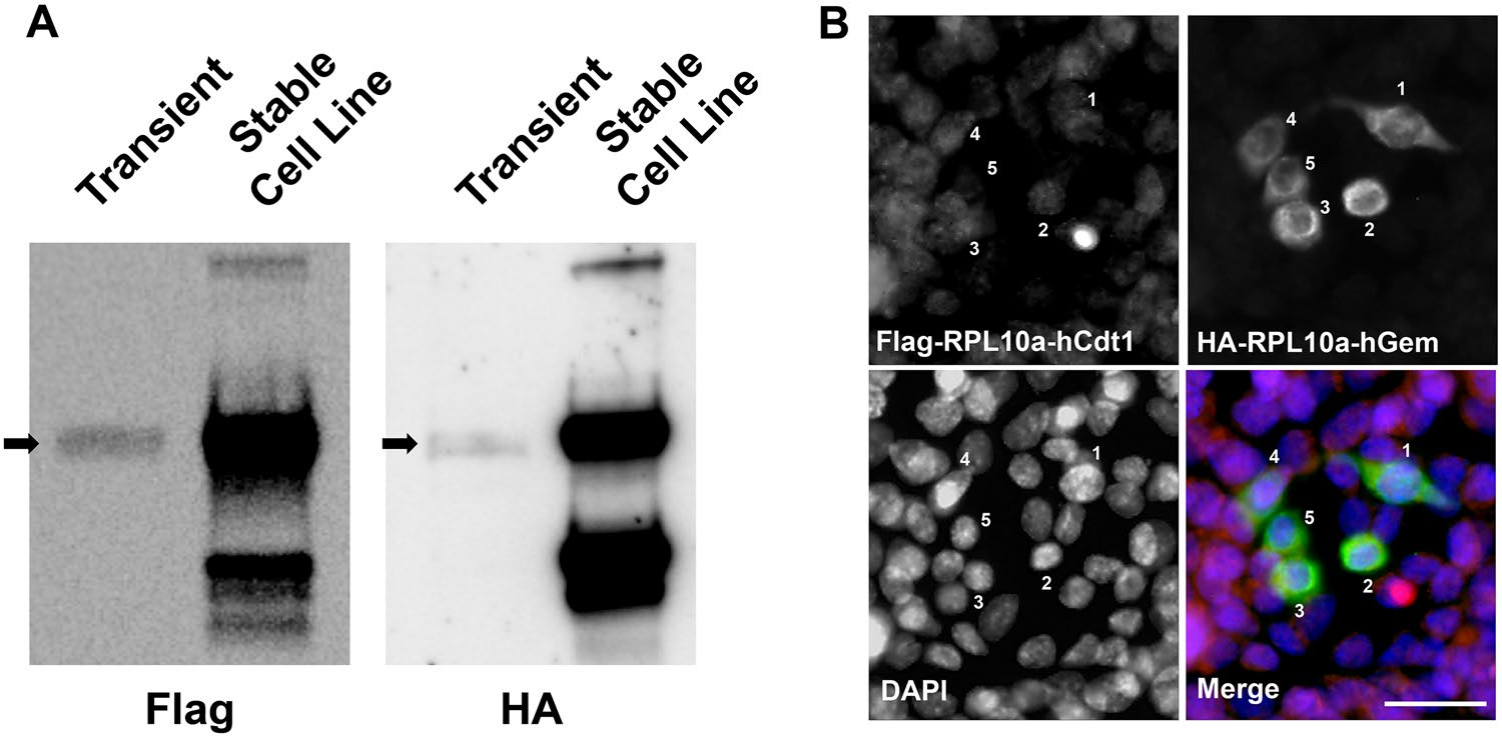
**(A)** Immunoblots of asynchronously cycling HEK293-T cells transiently transfected with ccTaggedRP and HEK293-T-FlpIn cells stably expressing ccTaggedRP probed with antibodies to Flag Tag or HA Tag. See Supplemental Fig. 7 for uncut blots. **(B)** Immunofluorescence of HEK293-T-FlpIn cells expressing ccTaggedRPs showing anti-Flag and anti-HA antibody statins, and DAPI. A merged image is shown. Cells denoted 1, 2, 3 and 4 express Flag-RPL10a-hCdt and HA-RPL10a-hGem, cell denote 5 expressed HA-RPL10a-hGem alone. All other cells express Flag-RPL10a-hCdt alone. Scale bar in the lower right corner corresponds to 20 μm.

To control for the copy number of recombinant DNA, we created the stable expression of ccTaggedRP through Flp-In insertion of a single copy of ccTaggedRP into HEK293-T cells (Fig. 2A). Immunofluorescent staining of Flag and HA identified distinct cells expressing Flag-RPLa10-hCdt alone, Flag-RPL10a-hCdt and HA RPL10a-hGem, and HA-RPLa10-hGem alone (Fig. 2b). The majority of cells were only positive for Flag-RPLa10-hCdt alone, consistent with G1 phase of the cell cycle.

**Figure 3:**
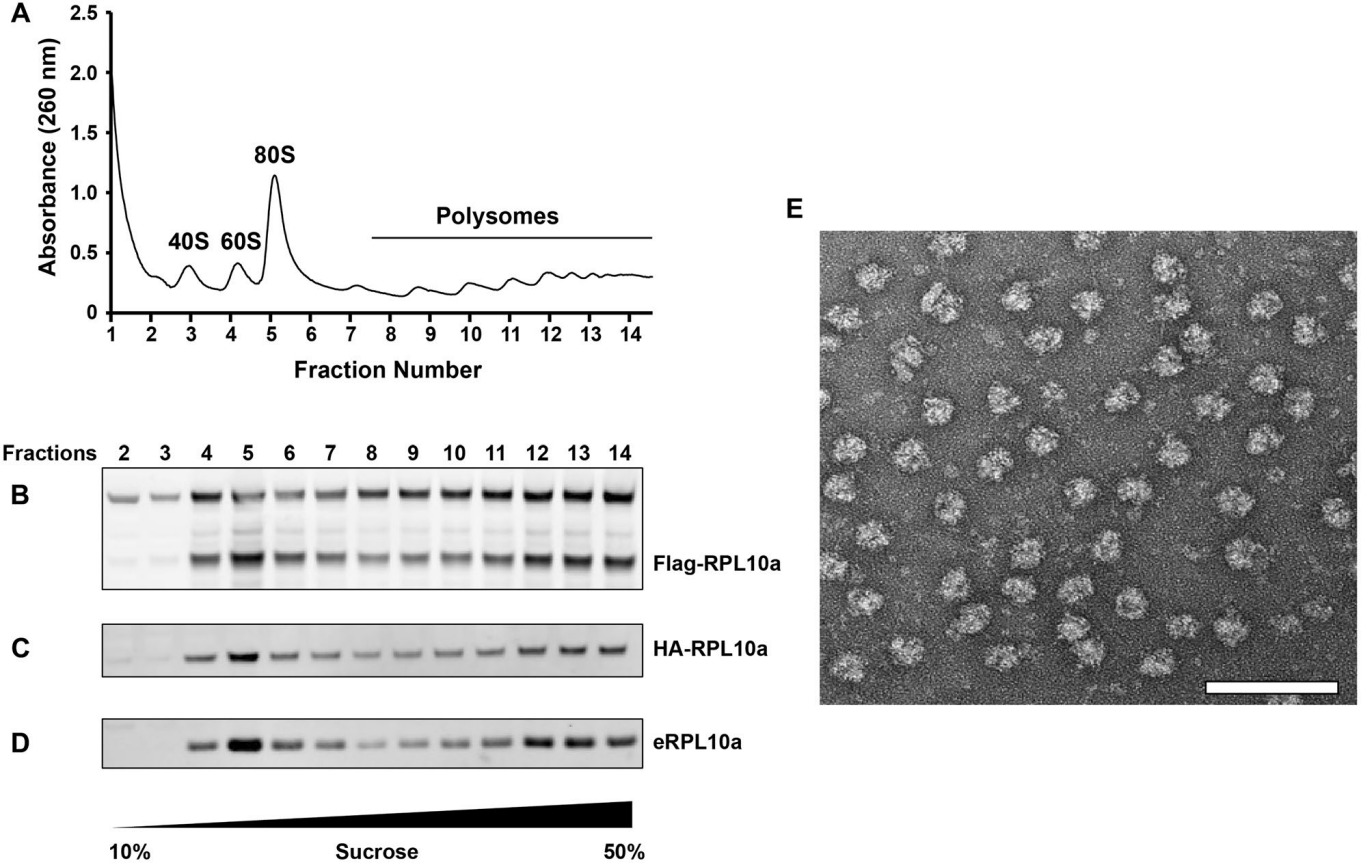
Ribosome analysis using sucrose gradient centrifugation of lysates from HEK293T-FlpIN cells stably expressing ccTaggedRPs. **(A)** Absorbance at 260 nm (A260) was measured during fractionation and showed distinct peaks corresponding to 40S and 60S subunits, 80S ribosomes, and polysomes. **(B–D)** Western blots corresponding to fractions from the sucrose gradient in panel A, using anti-Flag tag **(B),** anti-HA tag **(C),** and anti-RPL10a (denoted eRPL10a) **(D)** antibodies. (E) Negative stain electron microscopy showed enrichment of 80S ribosomes from fraction #5 of the corresponding A260 peak for 80S ribosomes. Scale bar is 100 nm. See Supplemental Fig. 9 for uncropped blots. Recombinant Flag-RPL10a-hCdt and HA-RPL10a-hGem were detected in fractions corresponding to the 60S ribosomal subunits, 80S ribosomes, and polysomes.

To evaluate the incorporation of recombinant ccTaggedRPs into ribosomes, we performed sucrose gradient centrifugation of lysates prepared from HEK-293 T cells stably expressing ccTaggedRPs. Cell lysates were fractionated on a 10% to 50% sucrose gradient while measuring the absorbance at 260 nm to detect ribosomal subunits (40S and 60S), in addition to 80S ribosomes, and translating ribosomes (polysomes) (Fig. 3A). Immunoblots of fractions identified the presence of recombinant Flag-RPL10a-hCdt and HA-RPL10a-hGem in the 60S large ribosomal subunits, 80S ribosomes, and polysomes, with a distribution similar to RPL10a. (Fig. 3B–D). Negative staining electron microscopy of fraction #5 demonstrated that 80S ribosomes were enriched in this fraction as expected (Fig. 3E). The findings support that Flag-RPL10a-hCdt and HA-RPL10a-hGem expressed from recombinant ccTaggedRP were incorporated into ribosomes and polysomes.

### ccTaggedRP expression changes during cell cycle synchronization

The HEK293-T Flp-In cells expressing ccTaggedRP were synchronized via serum starvation, which is known to increase the percentage of cells in G_0_/G_1_ as starvation persists. In accordance with expectations, the expression of Flag-RPL10a-hCdt recombinant protein was progressively increased with starvation, suggesting an enrichment in the Flag-RPL10a-hCdt construct in G_0_/G_1_ while the expression of HA-RPL10a-hGem recombinant protein progressively decreased, suggesting an enrichment in HA-RPL10a-hGem construct in G_2_/M. (Supplemental Fig. 1A, B). Specifically, compared to serum-fed HEK293-T Flp-In cells, HA-RPL10a-hGem expression decreased by 82% after 48 h of serum starvation. (Supplemental Fig. 1C).

**Figure 4:**
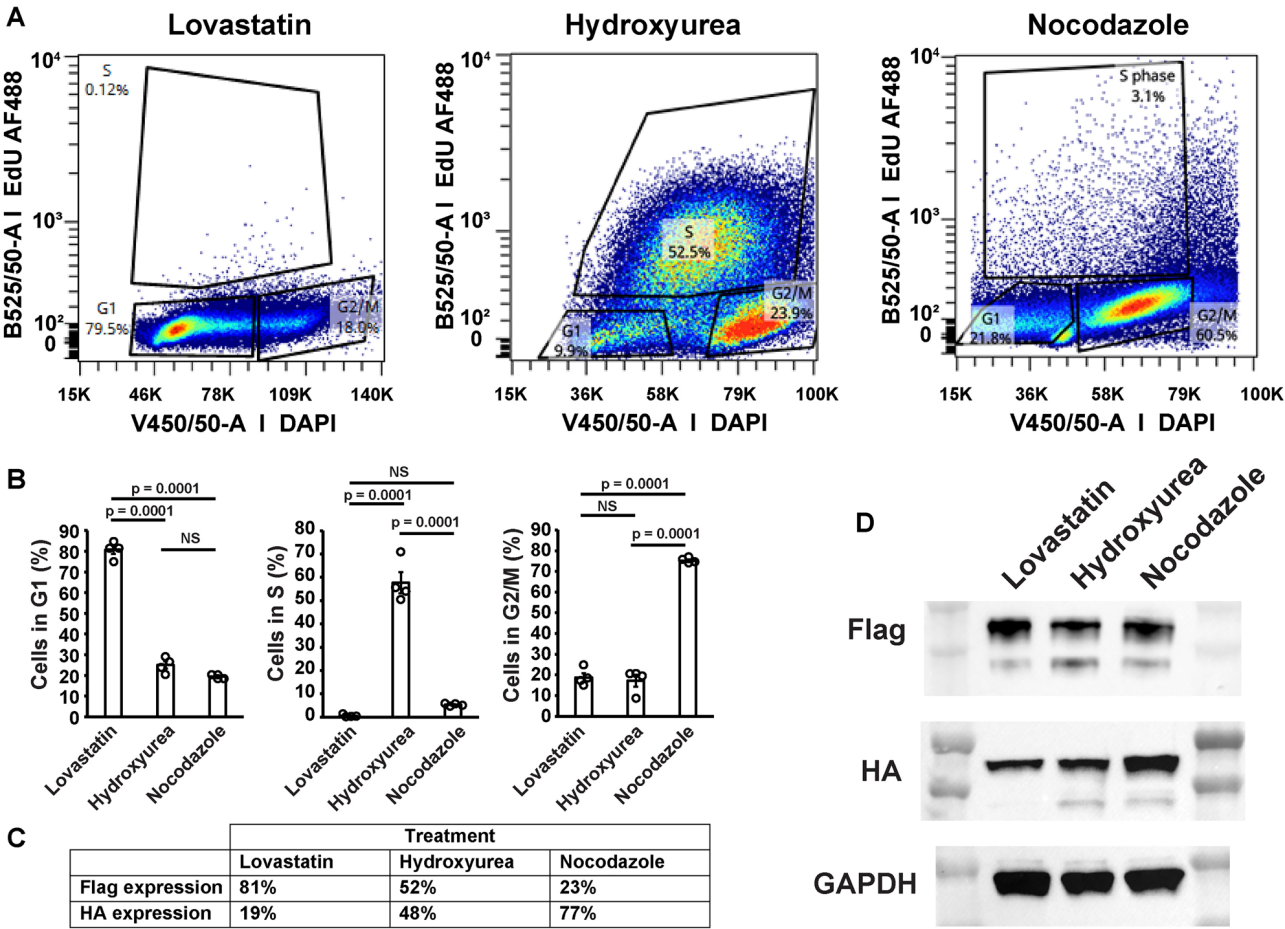
The expression of Flag-RPL10a-hCdt and HA-RPL10a-hGem is dependent on the phase of the cell cycle. **(A)** FACS quantitation of HEK293-T-Flp-In cells stably expressing ccTaggedRPs in G_1_, S, and G_2_/M treated with after synchronization with Lovastatin, Hydroxyurea or Nocodazole. The percentage of cells in G_1_, S, and G_2_/M under each treatment is shown. B525/50-A EdU AF488 represents EdU intensity and V450/50-A DAPI represents DNA content. These measurements were used to gate cells in G_1_, S, and G_2_M. **(B)** Quantitation of G_1_, S, and G_2_/M of cells treated with Lovastatin, Hydroxyurea, or Nocodazole. Values are mean ± SEM. P-values were calculated by ANOVA with Tukey test for multiple comparisons. N = 4 plates per condition. **(C)** Predicted relative expression of Flag and HA based on the results shown in panel B. **(D)** Western blots of Flag-RPL10a-hCdt (top), HA-RPL10a-hGem (middle), and GAPDH (bottom) corresponding to the cells treated with Lovastatin, Hydroxyurea, or Nocodazole and analyzed by FACS in panel A. See Supplemental Fig. 10 for uncropped blots.

To further validate the cell cycle dependence of Flag-RPL10a-hCdt and HA-RPL10a-hGem expression, HEK293-T Flp-In cells expressing ccTaggedRP were synchronized by treatment with Lovastatin, Hydroxyurea or Nocodazole and enrichment in G_1_, S, and G_2_/M were confirmed by fluorescent activated cell sorting (FACS) (Fig. 4A–C and Supplemental Fig. 2). Cells treated with Lovastatin were 80.7% G_1_, 0.5% S, and 18.8% G_2_/M. Hydroxyurea were 25.1% G_1_, 57.7% S, and 17.2% G_2_/M. Cell treated with Nocodazole were 19.5% G_1_, 5.2% S, and 75.3% G_2_/M (Fig. 4B). Based on the FACS data and predicted ideal behavior of the degrons with Flag expressed in G1 and S and HA expressed in S and G2/M, the relative changes in Flag and HA expression were estimated (Fig. 4C). Western blotting of cells treated with Lovastatin, Hydroxyurea, or Nocodazole showed reciprocal changes in the expression of Flag-RPL10a-hCdt and HA-RPL10a-hGem (Fig. 4d). Flag-RPL10a-hCdt was increased and decreased in expression in G1 and G2M phases, respectively. HA-RPL10a-hCdt was decreased and increased in expression in G1 and G2M phases, respectively.

### ccTaggedRP identifies genes expressed during G_2_-M in asynchronously cycling cells

**Figure 5:**
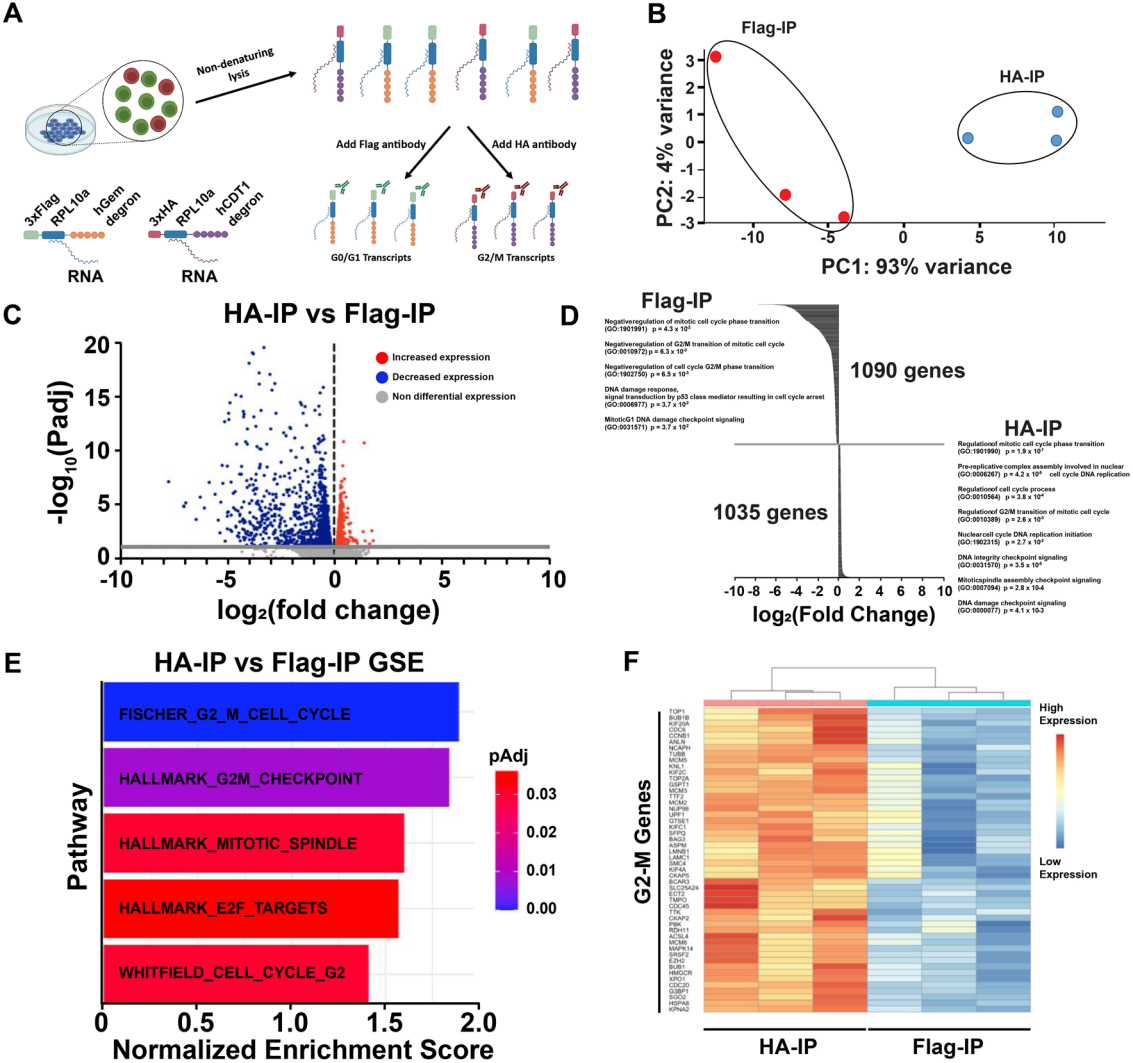
ccTaggedRP identifies genes expressed during G_2_-M in asynchronously cycling cells. **(A)** Schematic of the immunoprecipitation of 3xFlagTag-RPL10a-hCdt(30–120) and 3xHA-Tag-RPL10a-hGeminin(1–110) for the purification and sequencing of RNA from asynchronously cycling HEK293-T-Flp-In cells expressing ccTaggedRP. **(B)** Principal component analysis (PCA) plot of RNA sequencing of mRNAs from immunoprecipitated Flag recombinant protein (red dots) and HA recombinant protein (blue dots). **(C)** Volcano plot of RNA sequencing of mRNAs from immunoprecipitated Flag recombinant protein and HA recombinant protein. The gray line denotes an adjusted p-value of 0.05. Genes with increased and decreased expression are denoted by red and blue dots, respectively. **(D)** Waterfall plot of the 1090 genes enriched in the 3xFlagTag-RPL10a-hCdt(30–120) and 1035 genes enriched in the 3xHA-Tag-RPL10a-hGeminin(1–110) immunoprecipitated samples. GO Terms are denoted with adjusted p-values shown. **(E)** Gene set enrichment (GSE) analysis of RNAseq of mRNAs from immunoprecipitated Flag recombinant protein and HA recombinant protein. Adjusted p-vales are shown. **(F)** Heat map of the expression of the top 50 G_2_-M genes from mRNAs from immunoprecipitated Flag recombinant protein and HA recombinant protein.

Asynchronous HEK293-T Flp-In cells stably expressing ccTaggedRP were cultured to 60% confluency prior to non-denaturing lysis. Recombinant Flag-RPL10a-hCdt and HA-RPL10a-hGem proteins were then immunoprecipitated (IP) using HA or Flag antibodies, and their associated RNAs were recovered for bulk RNA-sequencing (Fig. 5A). Principal component analysis (PCA) displayed distinct clustering of the HA- and Flag-IP samples (Fig. 5B). This method resolved 2,125 differentially expressed genes with an adjusted p-value ≤ 0.05 between the HA- and Flag-IP RNA samples (Fig. 5C and Supplemental Table 1). Transcripts enriched in the HA-IPs were associated with regulation of the mitotic cell cycle and G_2_-M transition of the mitotic cell cycle while transcripts enriched in the Flag-IPs were associated with the negative regulation of both the mitotic cell cycle and the G_2_-M transition of the mitotic cell cycle (Fig. 5D). Additionally, Gene Set Enrichment (GSE) Analysis identified enrichment of G_2_-M cell cycle and checkpoint pathways in the HA-IPs (Fig. 5E). Furthermore, expression of G_2_-M genes was significantly increased in the HA-IPs compared to the Flag-IPs (Fig. 5F).

### scRNAseq of HEK293-T cells identifies populations of G_1_-S and G_2_-M

**Figure 6:**
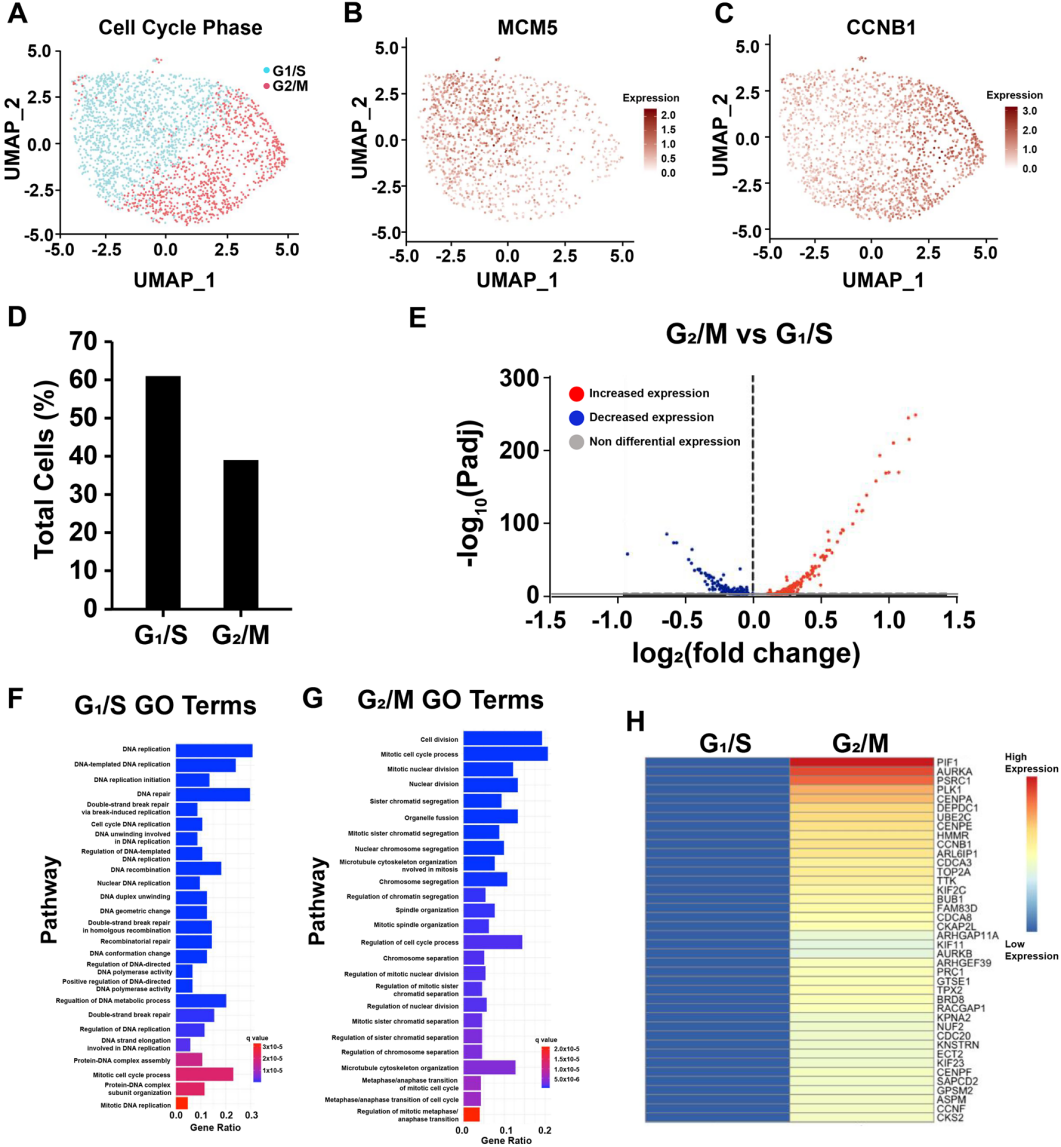
scRNAseq of HEK293-T cells identifies populations of G_1_-S and G_2_-M. **(A)** UMAP of cells assigned to G_1_/S and G_2_/M identity in HEK293-T cell culture. **(B,C)** UMAP of MCM5 and CCNB1 expression. **(D)** Percentage of total cells assigned to G_1_/S and G_2_/M identities. **(E)** Volcano plot of differential gene expression between G_1_/S and G_2_/M clusters. **(F,G)** Gene Ontology results via hypergeometric testing of genes enriched in the G_1_/S cluster and G_2_/M cluster**. (H)** Heat map of the average expression for the top 40 G_2_/M genes between the G_1_/S and G_2_/M clusters.

To further validate ccTaggedRP, scRNAseq data for HEK293-T cells were sourced from 10× Genomics for comparison of transcripts expressed during G_1_/S and G_2_/M. Cells within the dataset were assigned to either “G_1_/S” or “G_2_/M” identities based on gene expression (Fig. 6A and Supplemental Figs. 3 and 4, and Supplemental Table 2). G_1_/S cells exhibited increased expression of MCM5, a protein important in DNA synthesis and upregulated in G_1_/S while G_2_/M cells exhibited increased expression of CCNB1, a protein important in cell cycle division and upregulated in G_2_/M (Fig. 6B, C). In this dataset, G_1_/S cells and G_2_/M cells accounted for 62.5% and 37.5% of the total number of cells, respectively (Fig. 6D). Differential expression analysis between the G_1_/S and G_2_/M cells revealed 530 differentially expressed genes with an adjusted p-value ≤ 0.05 (Fig. 6E). Hypergeometric testing of the differentially expressed genes indicated an enrichment in DNA replication processes and mitotic cell cycle processes for cells in G_1_/S and G_2_/M respectively (Fig. 6F, G). Furthermore, G_2_/M cells displayed increased expression of G_2_-M genes compared to G_1_/S cells (Fig. 6H).

### Comparison of genes expressed in G_2_-M during asynchronous cycling from ccTaggedRP and scRNAseq

To investigate the differences between the two methodologies, functionalization of shared and method-specific differentially expressed genes was performed. Comparison of differentially expressed genes from the ccTaggedRP method and scRNAseq found 2019 genes specific to the ccTaggedRP method, 423 genes specific to the scRNAseq method, and 107 genes shared between the two (Supplemental Fig. 5A). Furthermore, hypergeometric testing of the shared differentially expressed genes indicated enrichment of genes associated with mitotic cell cycle processes (Supplemental Fig. 5B).

## Discussion

We sought to develop an extension of the RiboTrap/RiboTag technologies that would facilitate the characterization of transcribed genes during distinct cell cycle phases of asynchronously cycling cells. RiboTrap and RiboTag use an EGFP-tagged RPL10a ribosomal protein subunit or HA-tagged RPL22 ribosomal protein subunit, respectively, for translating ribosome affinity purification (TRAP) of mRNA populations and characterization of gene expression^2–4,24–27^. Both methods have provided new insights regarding the gene expression of specific cell types in complex tissues. For example, RiboTrap has been used to investigate the differential gene expression of excitatory neurons and interneuron subtypes^28^, interstitial/pericytes, myeloid cells, nephron cells, and endothelial cells^29^ while Ribotag has been used to characterize gene expression in neurons^3^, microglia^30^, oocyte maturation^31^, and cardiomyocyte and endothelial cells^4^ in diverse disease states. Notably, the gene expression is obtained from bulk RNA sequencing of pooled mRNAs, meaning that the total pool of expressed transcripts dilutes the cell cycle phase-specific transcripts and modifies interpretation. To overcome this limitation, we describe the first-generation ccTaggedRP technology based on merging aspects of FUCCI and RiboTag methods. FUCCI employs degrons that target proteins for degradation during the G_1_/S or G_2_/M phases of the cell cycle^22,23^. By creating ccTaggedRP, which contains different epitope-tagged RPL10a proteins with either hCdt1 or hGem1 degrons, the mRNAs expressed during cell cycle phases can be enriched by TRAP. The initial polypeptide is bicistronic and cleaved into distinct tagged RPL10a chimeric proteins whose subsequent abundance is dictated by the phase of the cell cycle via hCdt1- and hGem-mediated degradation. Additionally, the targeted insertion of a single copy of ccTaggedRP in HEK293-T-Flp-In cells avoided the effects of multiple copy insertion or insertion into a transcriptionally silenced region of the genome, which could adversely affect the expression of ccTaggedRP.

3xFlagTag-RPL10a-hCdt(30–120) and 3xHA-Tag-RPL10a-hGeminin were differentially expressed during G_1_/S and G_2_/M based on synchronization of different phases of the cycle using inhibitors, had similar subcellular locations as endogenous ribosomal proteins, were incorporated into the 60S ribosomal subunit, 80S ribosomes, and polysomes, and facilitated TRAP of mRNAs expressed during G_1_/S and G_2_/M. Bulk RNA sequencing of mRNAs associated with the immunoprecipitation of 3xHA-Tag-RPL10a-hGeminin identified transcripts enriched in the regulation of the mitotic cell cycle and G_2_-M transition of the mitotic cell cycle. Conversely, the immunoprecipitation of 3xFlagTag-RPL10a-hCdt(30–120) identified transcripts enriched in the negative regulation of the mitotic cell cycle and the G_2_-M transition of the mitotic cell cycle. Collectively, our data suggests the predominance of the HA recombinant protein and Flag recombinant protein in the G_2_/M and G_1_/S phases of the cell cycle, respectively.

Next, we compared the results of TRAP using ccTaggedRP with scRNAseq results of HEK293-T cells obtained by 10× Genomics. Cells from the scRNAseq were assigned to either G_1_/S or G_2_/M phases based on their expression profiles. The differential expression between the cell cycle clusters was then compared to those obtained by ccTaggedRP. A comparison of differentially expressed genes from the ccTaggedRP method and scRNAseq found 2019 genes specific to the ccTaggedRP method, 423 genes specific to the scRNAseq method, and 107 genes shared between the two methods. Evaulation of the shared differential gene expression confirmed similar gene expression using the two methods; however, the ccTaggedRP approach resolved nearly four-fold more differentially expressed genes between conditions. Moreover, the transcript enrichment from the ccTaggedRP method was similar if not greater than that of the scRNAseq method. Additionally, the significantly lower cost of bulk RNAseq and its consequent opportunity for multiple biological replicates further bolster the potential of ccTaggedRP as an alternative to scRNAseq for resolving cell cycle-specific gene expression.

There are, however, limitations to the ccTaggedRP technology. First, the exchange rates of RPLs into and out of ribosomes are likely different for each RPL and could affect the efficiency of the ccTaggedRP technology (Supplemental Fig. 6). For example, RPLs with slower exchange rates may not degrade efficiently during cell cycle phases and influence the interpretation of TRAP data. Therefore, future ccTaggedRPs that use different RPLs will require careful characterization, given the dynamic properties of RPLs. This problem may be further exacerbated for rapidly cycling tissue, in which there is insufficient time for degradation of the constructs between phases. FUCCI constructs encode fluorescently labeled degrons and may be more amenable to degradation compared to Flag-RPL10a-hCdt and HA-RPL10a-hGem that are incorporated into ribosomes, potentially slowing their degradation. However, HA-RPL10a-hGem demonstrated more pronounced expression changes in response to the cell cycle. As presented in this study, we were able to resolve cell cycle-specific gene expression in a highly replicative embryonic kidney epithelial cell line, suggesting that the kinetics of the construct are sufficiently fast to support analyses in most contexts. Second, the heterogeneity of ribosomes could influence the interpretation of TRAP data^32^. Third, we acknowledge the presence of lower molecular weight HA and FLAG recombinant proteins, which are likely products of protein degradation. This is to be expected as each of the recombinant proteins have C-terminal degrons. Importantly, despite the presence of these degradation products, cell cycle-specific gene expression was still able to be discerned. Finally, we tested the technology in asynchronous cells in culture, a scenario that provided adequate signal detection. Translating the ccTaggedRP technology to a transgenic mouse for in vivo experiments may be challenging.

Despite these limitations, the ccTaggedRP method is promising and has great potential to identify cell cycle phase-specific gene expression in complex tissues. Moreover, it can be combined with other technologies to create even more powerful approaches. The ccTaggedRP construct could be inserted downstream of tissue-specific promoters to resolve cell cycle-specific gene expression in specific tissues. This may be particularly useful due to the high degree of contamination observed in other purification methods. Furthermore, in tissues with low replicative capacities, this methodology affords the ability to pool multiple biological samples to characterize these sparse populations. Alternatively, the methods could be adapted to create chimeras of epitope-tagged Argonaut proteins containing hCdt1 and hGem to survey the expression of microRNAs expressed during G_1_/S and G_2_/M in asynchronous cycling cells in complex tissues.

## Methods details

The Flp-In HEK293-T cells stably expressing ccTaggedRP cell line and plasmid are available upon reasonable request to the corresponding author. The CAG-ccTaggedRP plasmid will be deposited into Addgene.

### Creation of the ccTaggedRP construct

A DNA construct encoding 3× Flag-Tag, human Cdt1 (amino acid 30 to 120), T2A, 3× HA-Tag, and human Geminin (hGem (amino acids 1 to 110)) and containing 5P BglII and 3P NheI restriction sites was synthesized by IDT DNA, Inc. The hCdt1 and hGem DNA sequences were similar to the sequences used in Fucci2aR^22^. CMV promoter and multicloning sites were removed from pSF-CAG-Kan (plasmid OGS505, Sigma, Inc) using BglII and NheI and replaced with the sequence containing 3× Flag Tag, hCdt1(30–120), T2A, 3× HA Tag, and hGem(1–110). The DNA sequence corresponding to cDNA of mouse RPL10a was synthesized by IDT DNA, Inc, and modified to add 5P and 3P AvrII or 5P and 3P MluI restriction sites. The add 5P and 3P AvrII or 5P and 3P MluI RPL10a cassettes were subcloned into 3× Flag Tag, hCdt1(30–120), T2A, 3× HA Tag, and hGem(10–110) to create in frame 3× Flag Tag-RPL10a-Cdt1(30–120)-T2A-3× HA Tag-RPL10a-hGem(10–110) in the pSF plasmid. Then, a CAG promoter was subcloned into the 3× Flag Tag-RPL10a-hCdt1(30–120)-T2A-3× HA Tag-RPL10a-hGem(10–110) plasmid to create the CAG-ccTaggedRP construct. We made the Flp-In HEK293-T cells stably expressing ccTaggedRP using the ccTaggedRP DNA construct that subcloned into a plasmid modified to contain a CAG promoter and 3P FRT and Hygromycin sequences corresponding to pcDNA5/FRT (ThermoFisher, Inc.). The Flp-In HEK293-T cells were purchased from Invitrogen, Inc and the Flp-In HEK293-T cells stably expressing ccTaggedRP were made according to the manufacture’s protocol. The DNA sequences of all constructs were validated by Sanger sequencing.

### Polysome profiling

Polysome profiling of HEK293T cells was conducted as described previously^33^. A 100 mg cell pellet was lysed with 500 µL of lysis buffer (20 mM HEPES–KOH pH 7.7, 100 mM KCl, 5 mM MgCl_2_, 0.5% IGEPAL, 1 mM DTT, 0.5× protease inhibitor cocktail (Promega), 4 units/mL RNase inhibitor (Promega), 100 µg/mL cycloheximide). The lysate was passed through a 23G needle four times and centrifuged at 11,200×*g* at 4 °C, for 8 min. The clarified lysate was loaded on top of a linear sucrose gradient (10–50% sucrose, 20 mM HEPES–KOH pH 7.7, 100 mM KCl, 10 mM MgCl_2_, 1 mM DTT). The sample was centrifuged at 230,000×*g* at 4 °C, for 2.5 h using an SW40 rotor. After centrifugation, a BioComp gradient profiler was used to fractionate the sucrose gradient at 0.2 mL/min into 800 µL fractions. The absorbance at 260 nm was measured during fractionation.

### Negative stain EM

For negative stain electron microscopy, a 10 µL sample corresponding to the 80S ribosome peak (fraction 5) was applied directly after fractionation to a glow discharged 400-mesh grid (EMS, CF400-Cu-50) and incubated for 5 min at room temperature. The grid was stained with 2% uranyl acetate twice for 1 min each. Excess fluid was blotted away with Whatman filter paper after each incubation period (Cytiva, 1001-090). Images were collected on an FEI F20 electron microscope operated at 120 kV and equipped with a TVIPS TemCam XF416 camera. Images were taken at a magnification of 62,000×, and a pixel size of 0.17 nm.

### Western blotting

For Western Blotting of sucrose gradient fractions, TCA (100% w/v) was added to each fraction at a final concentration of 10% and the samples were incubated for 30 min on ice. The samples were centrifuged at 13,000×*g* at 4 °C for 15 min. Pellets were washed with ice cold acetone and centrifuged at 13,000 xg for 10 min (2×). The pellets were dried, then resuspended in 100 µL of 1× SDS-PAGE sample buffer (50 mM Tris–HCl pH 6.8, 2% SDS, 6% glycerol, 0.004% bromophenol blue, 1% β-mercaptoethanol). Proteins were resolved on a NuPAGE 4–12% Bis–Tris minigel (Invitrogen, NP0323BOX) and transferred onto a 0.2 µm PVDF membrane (Invitrogen LC2002). The membrane was blocked in 3% BSA in 1× PBST (0.1% Tween-20) and probed with FLAG (Sigma, F1804), HA (Invitrogen, 26183), and RPL10a (Invitrogen, MA5-44710) antibodies diluted in 1% BSA in 1× PBST. Secondary anti-mouse (Invitrogen, A21058) and anti-rabbit (LI-COR, 926-32213) antibodies were used for detection with the LI-COR Odyssey imager.

For Western blotting of cell lysates, HEK293-T cells expressing ccTaggedRP were cultured in DMEM (CAT#:11965-092, ThermoFisher) supplemented with 10% fetal bovine serum (CAT#: 10438026, ThermoFisher) and 500 µg/mL Hygromycin B (CAT#: 10687010, ThermoFisher) until 80% confluency. Cells were washed once with Dulbecco's phosphate-buffered saline (DPBS) (CAT#: 14040133, ThermoFisher) and lysed in RIPA buffer (CAT#: 89900, ThermoFisher) supplemented with protease inhibitors (Roche, 04693159001). Protein concentration was quantified with the Pierce BCA kit (CAT#: 23225, ThermoFisher) and normalized. 4× loading buffer was added to samples. 25 µg of total protein was run on SDS gels (CAT#: 4561034, Bio-rad) and transferred to PVDF membranes with 45 µm pores (CAT#: 84-895, Prometheus). The membranes were blocked in 5% non-fat milk (Great Value) in Tris-buffered saline with 0.1% Tween 20 (TBST). Membranes were then incubated overnight with primary antibodies against FLAG (1:1000, CAT#: F1804, Invitrogen), HA (1:1000, CAT#: 26183, Invitrogen), or GAPDH (1:1000, CAT#: ab9485, Abcam) in 5% milk solution. Membranes were washed three times with TBST and subsequently incubated in TBST with HRP-conjugated secondary antibodies (CAT#: G-21234, Invitrogen). Membranes were washed twice with TBST and once with TBS. Chemiluminescent reaction was catalyzed by addition of 1:1 1 Ambersham ECL Detection Kit. Membranes were imaged on a Bio-Rad ChemiDoc Touch Imaging System (Bio-Rad, Inc.). Densitometry was quantified using Bio-Rad Image Lab 6.1 (Rio-Rad, Inc.).

### Serum starvation of cultured cells

HEK293-T cells expressing ccTaggedRP were cultured in DMEM (CAT#:11965-092, ThermoFisher) supplemented with 10% fetal bovine serum (CAT#: 10438026, ThermoFisher) and 500 µg/mL Hygromycin B (CAT#: 10687010, ThermoFisher) until 80% confluency. Starved samples were then cultured in DMEM supplemented with only fetal bovine serum for the indicated amount of time.

### Pharmacologic synchronization of cultured cells

HEK293-T cells expressing ccTaggedRP were cultured in Dulbecco's Modified Eagle Medium (DMEM) (CAT#:11965-092, ThermoFisher) supplemented with 10% fetal bovine serum (CAT#: 10438026, ThermoFisher) and 500 µg/mL Hygromycin B (CAT#: 10687010, ThermoFisher) until 70% confluency. Cells were then treated with vehicle, 100 μM Lovastatin (CAT#: 400046, Sigma) for 48 h, 20 µg/ml hydroxyurea (CAT#: 400046, Sigma) for 24 h, or 5 µg/ml nocodazole (M1404, Sigma) for 48 h.

### Flow cytometry

Cells were incubated with 10 µM EdU for 1.5 h prior to harvest. Following harvest, cells were incubated with fixable LIVE/DEAD stain (CAT#: L34992, ThermoFischer) for 20 min in the dark and then washed with PBS. Following staining, cells were fixed with 4% paraformaldehyde (PFA) for 20 min and then were washed with 1% BSA. Cells were permeabilized and underwent the Click-it reaction according to manufacturer's instructions (CAT#: C10633, ThermoFischer). Cells were then washed with permeabilization buffer and stained with FxCycle Violet (CAT#: F10347, ThermoFischer) for 1 h in the dark. Data was obtained with a BD LSR Fortessa and analyzed with OMIQ (Dotmatics). Gating strategy is illustrated in Supplemental Fig. 2.

### Immunofluorescence of cultured cells

HEK293-T cells expressing ccTaggedRP were cultured in DMEM (CAT#:11965-092, ThermoFisher) supplemented with 10% fetal bovine serum (CAT#: 10438026, ThermoFisher) and 500 µg/mL Hygromycin B (CAT#: 10687010, ThermoFisher) until 80% confluency. Medium was removed, and cells were fixed in 4% PFA. Cells were washed and incubated overnight in 1× PBS. Slides were then incubated at 90 °C in 1× unmasking solution (CAT#: H-3300, Vector Labs). Slides were cooled to room temperature and then washed three times with 1× PBS. Slides were blocked in 1× PBS containing fish skin gelatin oil (FSG) and Donkey Serum (CAT#: S30-100ML, Millipore Sigma) for 1 h at room temperature. Cell slides were incubated in primary antibodies against FLAG (CAT#: PAB29056, Abnova) and RPL10a (CAT#: PA5-62845, Invitrogen) in 1× PBS contacting FSG overnight at 4 °C. Slides were washed once with 1× PBS containing FSG and 0.1% Tween-20 and once with 1× PBS. Cells were then incubated with secondary antibodies against donkey anti-chicken (CAT#: 703-585-155, Jackson ImmunoResearch) and donkey anti-rabbit IgG (CAT#: 711-475-152, Jackson ImmunoResearch) in 1× PBS containing FSG and 0.1% Tween-20. Slides were washed once with 1× PBS containing FSG and 0.1% Tween-20 and once with 1× PBS. Cover slips were then mounted with VECTASHIELD^®^ Antifade Mounting Medium with DAPI (H-1200-10, Vector Laboratories).

### Cell culture, lysis, immunoprecipitations, and RNA recovery

HEK293-T cells expressing ccTaggedRP were cultured in Dulbecco's Modified Eagle Medium (DMEM) (CAT#:11965-092, ThermoFisher) supplemented with 10% fetal bovine serum (CAT#: 10438026, ThermoFisher) and 500 µg/mL Hygromycin B (CAT#: 10687010, ThermoFisher) until 70% confluency. Cells were washed with DPBS (CAT#: 14040133, ThermoFisher) and lysed in Pierce™ IP Lysis Buffer (CAT#: 87787, ThermoFischer) supplemented with 1× protease inhibitor (CAT#: 87785, ThermoFischer) and RNAse Inhibitor (CAT#: AM2682, Invitrogen). Lysed samples were incubated overnight with 5 µg of IP antibodies against FLAG (CAT#: F1804, Invitrogen) or HA (CAT#: 26183, Invitrogen). 0.25 mg of Pierce Protein A/G Magnetic Beads (CAT#: 88802, ThermoFischer) were added, and the resulting mixture was incubated overnight with rocking at 4 °C. Magnetic bead-target complexes were collected with a magnetic stand, and the remaining solution was aspirated. Samples were washed twice with IP lysis buffer and resuspended in RW1 buffer. RNA was then isolated with the RNeasy mini kit (CAT#: 74104, Qiagen) according to the manufacturer's instructions. Isolated mRNA samples were quantified and submitted to Genewiz for further processing and sequencing.

### RNA sequencing analyses

cDNA library preparation with adapter ligation, and sequencing using an Illumina® HiSeq® system were performed by Genewiz, Inc. Reads underwent quality control, trimming, alignment, and quantification according to Genewiz standard workflows. Sequence reads were trimmed to remove possible adapter sequences and nucleotides with poor quality using Trimmomatic v.0.36. The trimmed reads were mapped to the *Homo sapiens* GRCh38 reference genome available on ENSEMBL using the STAR aligner v.2.5.2b. The STAR aligner is a splice aligner that detects splice junctions and incorporates them to help align the entire read sequences. BAM files were generated as a result of this step. Below are the statistics of mapping the reads to the reference genome. Genes with less than ten reads in at least three samples (the minimum sample size for a group) were excluded for subsequent analysis. Differential expression analysis was performed using R and the DEseq2 package. Functionalization was performed using ClusterProfiler package in R and GSEA software.

### scRNAseq analysis

A scRNAseq dataset of cultured HEK293-T cells was sourced from 10× Genomics (10× Genomics). Sequencing yielded 737,280 cells with an average of 66.58 reads per cell and 19.45 genes per cell. Poor quality cells were removed, using the following inclusion criteria: cells with greater than 2000 genes, greater than 7500 reads, and less than 7.5% mitochondrial DNA. Cells were assigned a “G_2_/M” or “G_1_/S” identity using the CellCycleScoring algorithm in Seurat and previously published data sets (supplemental Fig. 7)^34,35^. Cells unable to be assigned confidently to either identity were discarded before downstream analysis. After filtering, 2447 cells remained with an average of 15,651.61 reads per cell and 3485.21 genes per cell. Data was normalized and scaled with the Seurat package in R. UMAP dimensionality reduction was performed with 11 principal components and a resolution of 0.5. Differential expression analysis based on the cell cycle cluster was performed using the Seurat package in R. Functionalization was performed using clusterProfiler package in R.

### Quantification and statistical analysis

Shapiro Wilk test was performed on continuous variables to evaluate the data distribution. Normally-distributed data and non-normally distributed data were then analyzed for statistical significance by Student t test and Mann–Whitney U test, respectively. Statistical significance for RNAseq and scRNAseq data was determined through Benjamini–Hochberg procedure and reported as the adjusted p-value. All data is presented as mean ± standard deviation.

### Inclusion and diversity

We support inclusive, diverse, and equitable conduct of research.

### Supplementary Information

Supplementary Legends.

Supplementary Table 1.

Supplementary Table 2.

Supplementary Figures.

## Data Availability

All data reported in this paper will be shared upon request. Please contact the corresponding author, M.J.W., for access to data. The RNAseq data files have been deposited into Gene Expression Omnibus (GEO), Accession ID: GSE225385 (https://www.ncbi.nlm.nih.gov/geo/query/acc.cgi?acc=GSE225385). This paper does not report original code. Any additional information required to reanalyze the data deposited in this paper is available from the lead contact upon request.
