# Supplementary material for: Cell cycle specific, differentially tagged ribosomal proteins to measure phase specific transcriptomes from asynchronously cycling cells
Source: Sci Rep. 2024 Jan 18;14:1623. doi: 10.1038/s41598-024-52085-5 (PMC10796924; doi:10.1038/s41598-024-52085-5)
Supplement: Supplementary file 1 — Supplementary Legends. [file 41598_2024_52085_MOESM1_ESM.docx]

**Supplemental Figure Legends**

**Supplemental Figure 1. (A)** Immunoblots of serum starved HEK293T-FlpIN cells stably expressing ccTaggedRP probed with antibodies to Flag-Tag or HA-Tag. See Supplemental Figure11 for uncut blots. **(B and C)** Immunoblots and quantitation of HA recombinant protein expression in serum fed conditions and after 48 hours of serum starvation. Opens bars show the means +/- SEM. Open circles are individual experiments. (N = 4) *P=0.012 by t-test. See Supplemental Figure 11 for uncut blots.

**Supplemental Figure 2.** Gating strategy for FACS of HEK293T FlpIn cells stably expressing ccTaggedRP. **(A)** Live cells sorting, **(C)** Singlet cells based on DAPI, and **(C)** EdU intensity to gate G1, S, and G2/M cells.

**Supplemental Figure 3.** G1/S and G2/M Clustering gene sets

**Supplemental Figure 4.** Quality-control filtering of scRNAseq data

**Supplemental Figure 5.** Comparison of genes expressed in G2-M during asynchronous cycling from ccTaggedRP and scRNAseq. **(A)** Venn diagram of differentially expressed genes found in the ccTaggedRP RNAseq dataset and the scRNAseq dataset. **(B)** Gene Ontology results via hypergeometric testing of shared differentially expressed genes between the two datasets.

**Supplemental Figure 6.** Possible mechanism for the incorporation and turnover of ccTaggedRPs into ribosomes

**Supplemental Figure 7.** Original uncropped western blots of Flag, HA, and RPL10a used in Figure 1C. Lanes 1-3 correspond to HEK293T cells transfected with empty vector, HEK293T cells transfected with ccTaggedRP construct, and non-transfected HEK293T cells, respectively. Red arrows denote recombinant protein.

**Supplemental Figure 8.** Original uncropped western blots of Flag and HA used in Figure 2A for the transiently transfected and stable cell lines. Lanes 1-2 correspond to HEK293T cells transiently transfected ccTaggedRP construct and HEK293T cells stably expressing ccTaggedRP, respectively. High exposure for HA was used to show the presence of HA recombinant protein in the transiently transfected cells. Red arrows denote recombinant protein.

**Supplemental Figure 9.** Original uncropped western blots of Flag, HA, and RPL10a used in Figure 3, B, C, and D. **(A)** anti-Flag, **(B)** Anti-HA, and **(C)** anti-RPL10a. Fractions numbers from sucrose gradient chromatography of HEK293T-FlpIn cells expressing ccTaggedRP are shown. Panel A shows a band at ~70 kD in all lanes that represents a possible artifact band or full-length ccTaggedRP that has not undergone cleavage at the internal T2A site.

**Supplemental Figure 10.** Original uncropped Western blots of HA and GAPDH used in Figure 4C. Lanes 1-3 correspond to HEK293T cells stably expressing ccTaggedRP and treated with (1) Lovastatin, (2) Hydroxyurea, or (3) Nocodazole. Red arrows denote Flag (Left), HA (Center), and GAPDH (right).

**Supplemental Figure 11.** Original uncropped western blots of Flag and HA used in Supplemental Figure 1A for serum starved cell. Lanes 1-5 correspond to HEK293T cells stably expressing ccTaggedRP after 16 20, 24, 36, and 48 hours if serum starvation, respectively. Lane x was not used for analyses. Red arrows denote recombinant protein.

**Supplemental Figure 12.** Original uncropped western blots of HA and GAPDH used in Supplemental Figure 1B. Lanes 1-4 correspond to HEK293T cells stably expressing ccTaggedRP cultured in serum-sufficient media and lanes 5-8 correspond to HEK293T cells stably expressing ccTaggedRP cultures in serum-depleted media. The red arrow denoted recombinant protein.

**Supplementary tables**

**Supplemental Table 1.** Excel file of differentially expressed genes identified by the immunoprecipitations of recombinant Flag-RPL10a-hCdt and HA-RPL10a-hGem proteins expressed in asynchronous HEK293-T Flp-In cells stably expressing ccTaggedRP. Ensembl Gene ID, counts in triplicateds of Flag-RPL10a-hCdt IP and triplicates of HA-RPL10a-hGem IP are shown. The log2 fold change, and adjusted p-values are listed.

**Supplemental Table 2.** Excel file of differentially expressed genes between G1/S and G2/M cells identified by single cell RNAseq. G1/S and G2/M are average relative transcript expression for the specified cell clusters. The log2 fold change, and adjusted p-values are listed.
