# Supplementary material for: Cell cycle specific, differentially tagged ribosomal proteins to measure phase specific transcriptomes from asynchronously cycling cells
Source: Sci Rep. 2024 Jan 18;14:1623. doi: 10.1038/s41598-024-52085-5 (PMC10796924; doi:10.1038/s41598-024-52085-5)
Supplement: Supplementary file 4 — Supplementary Figures. [file 41598_2024_52085_MOESM4_ESM.docx]

**Supplemental Figure 1**

**
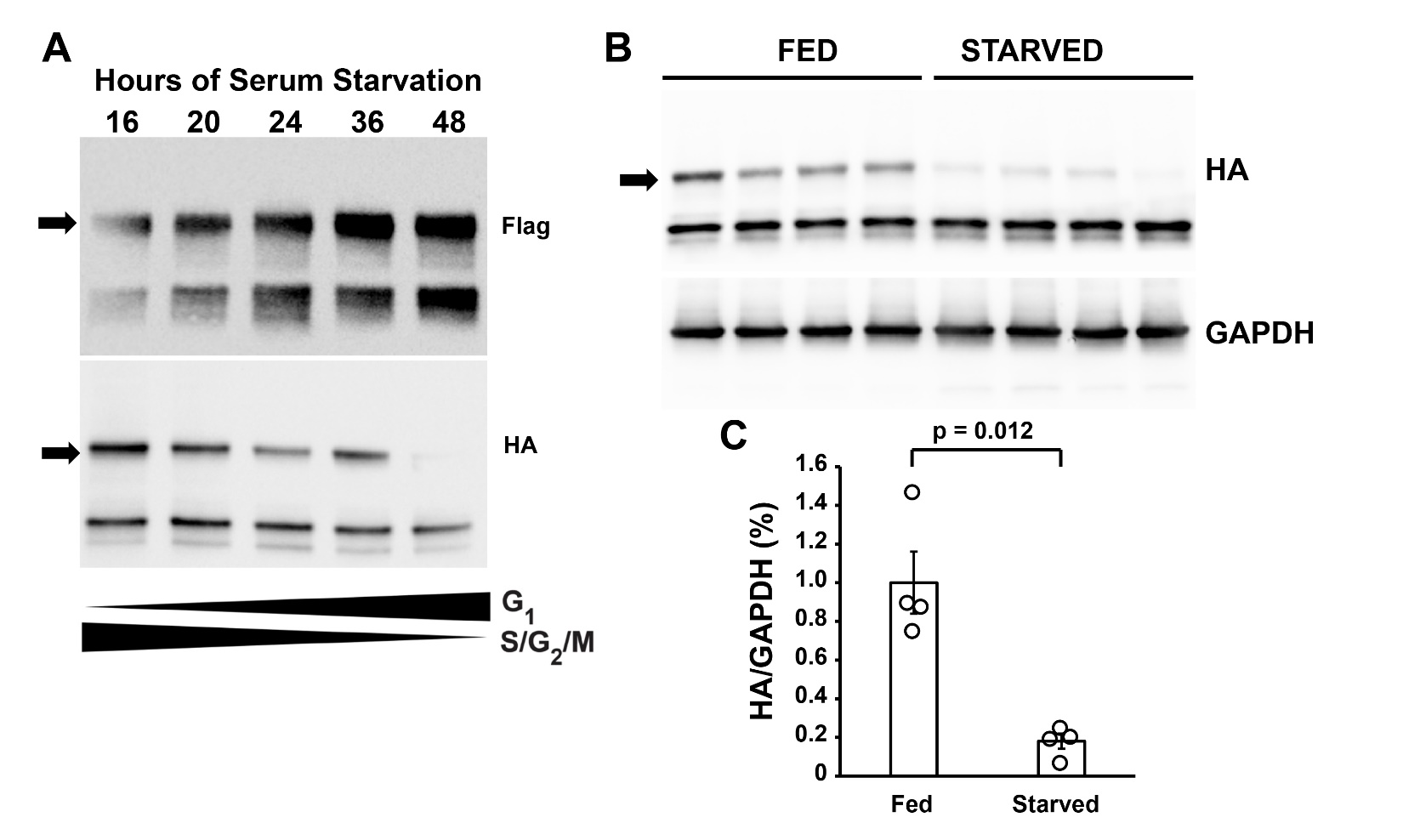
**

**
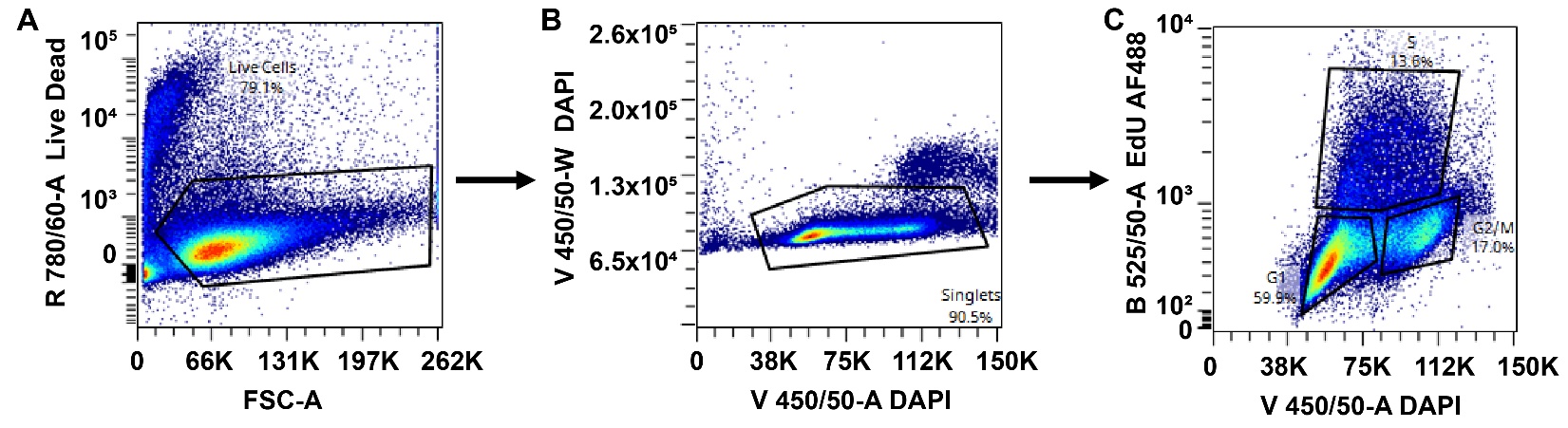
Supplemental Figure 2**

**Supplemental Figure 3**

**
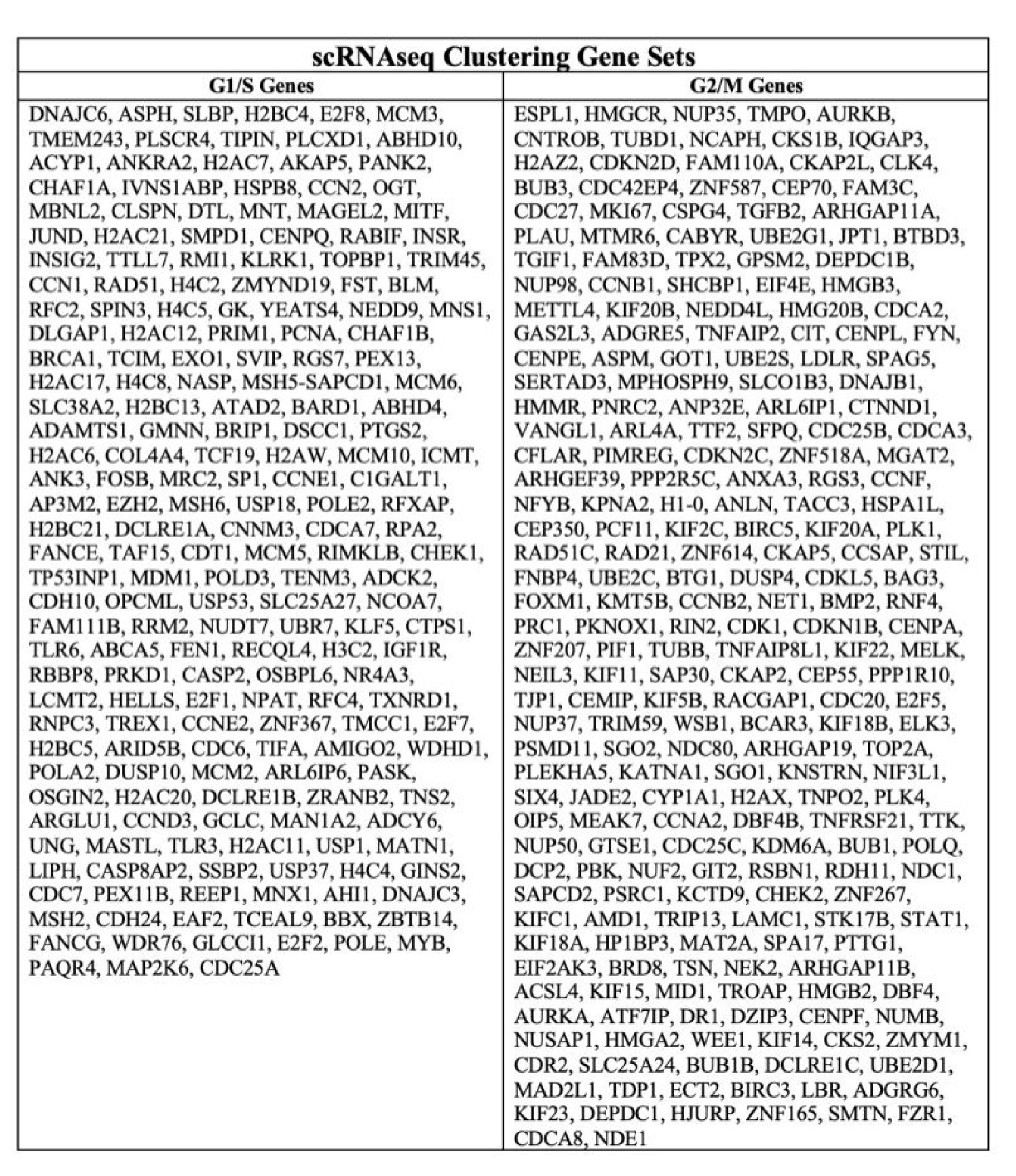
**

**Supplemental Figure 4**

**
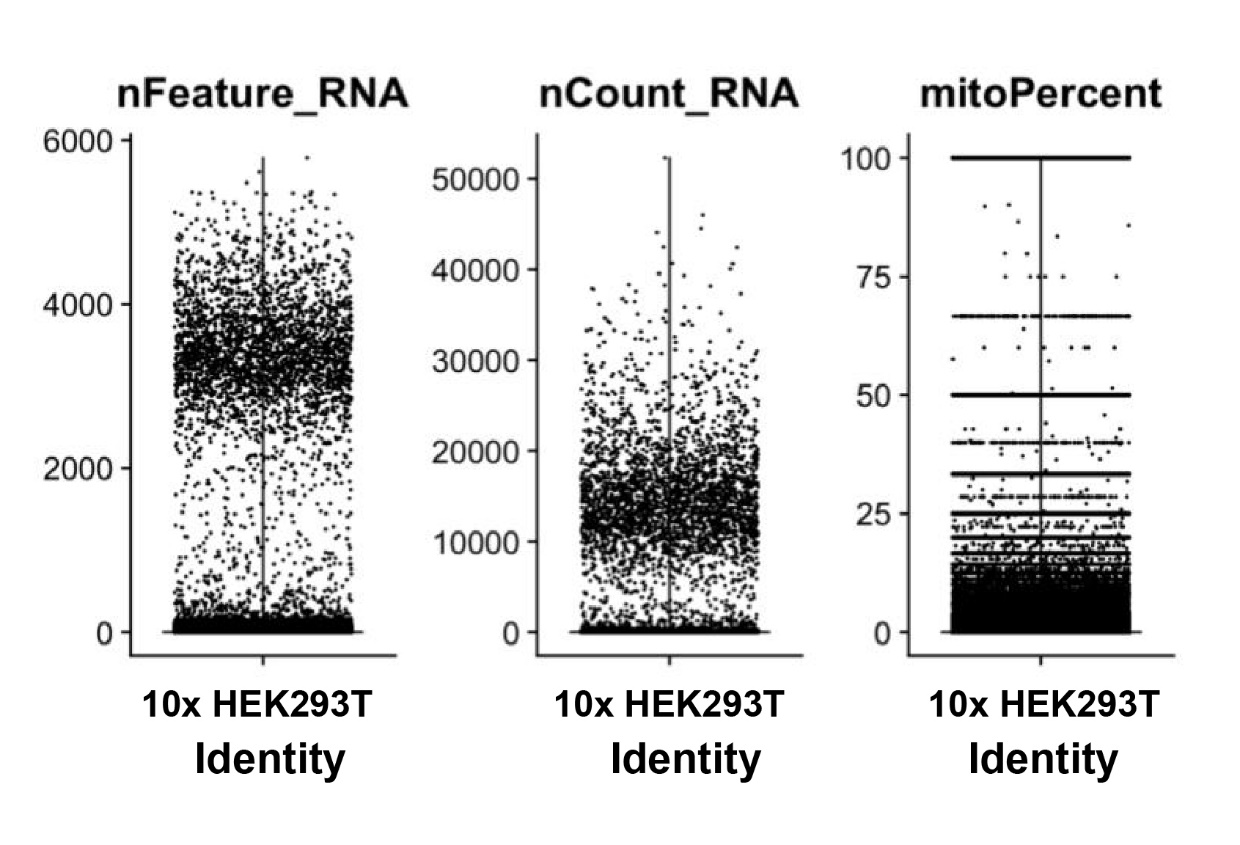
**

**Supplemental Figure 5**

**
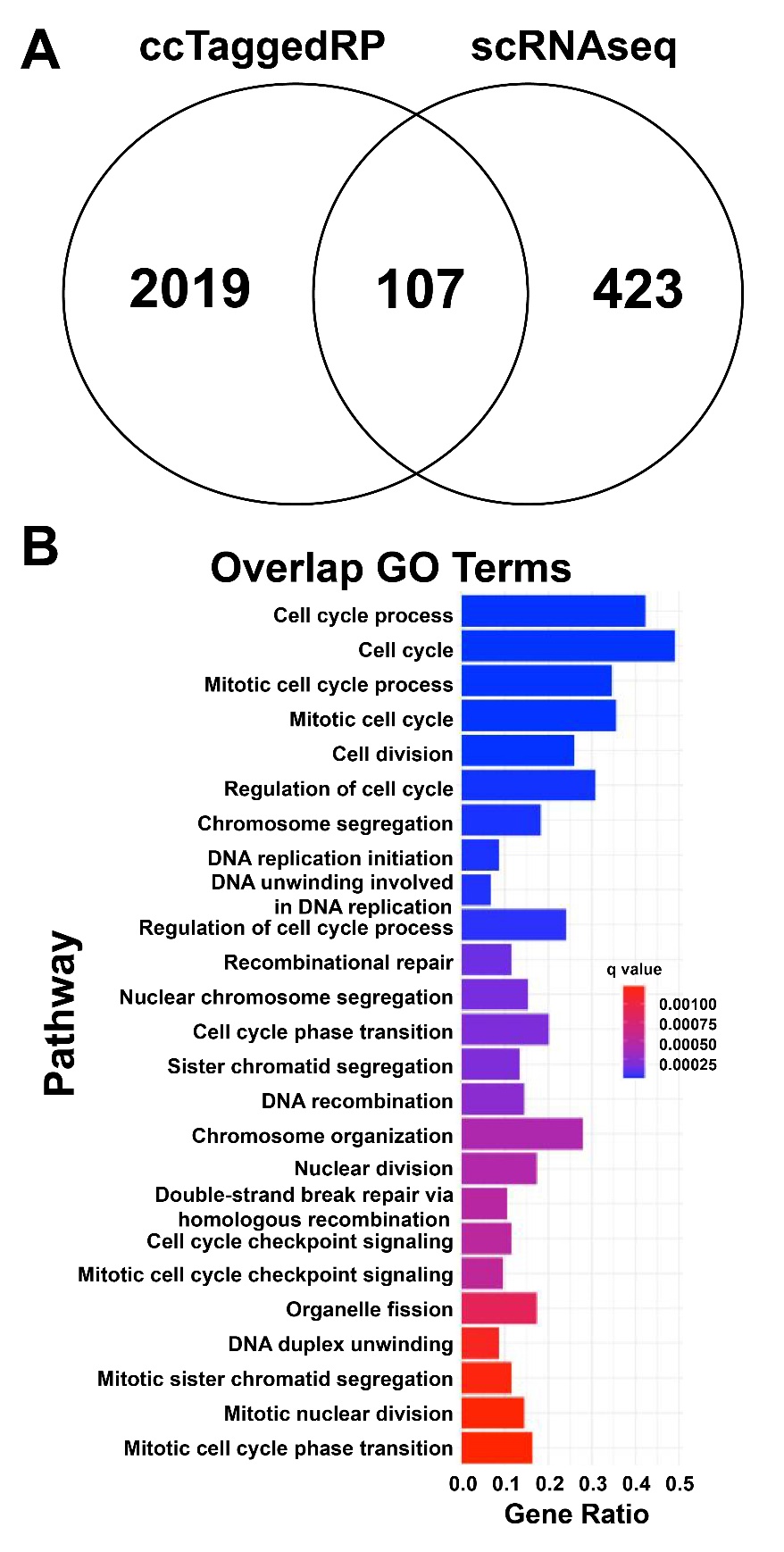
**

**Supplemental Figure 6**

**
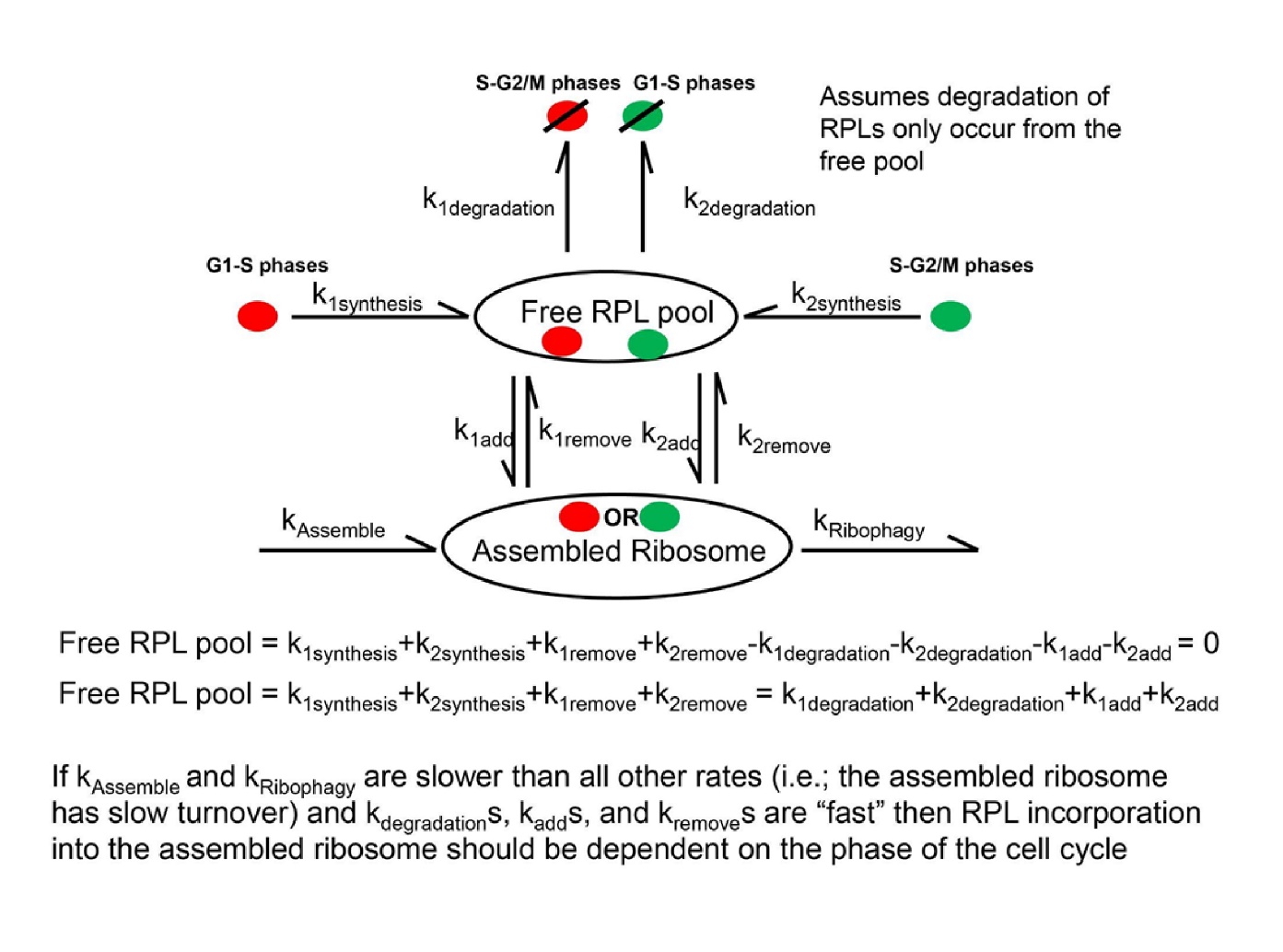
**

**Supplemental Figure 7**

**
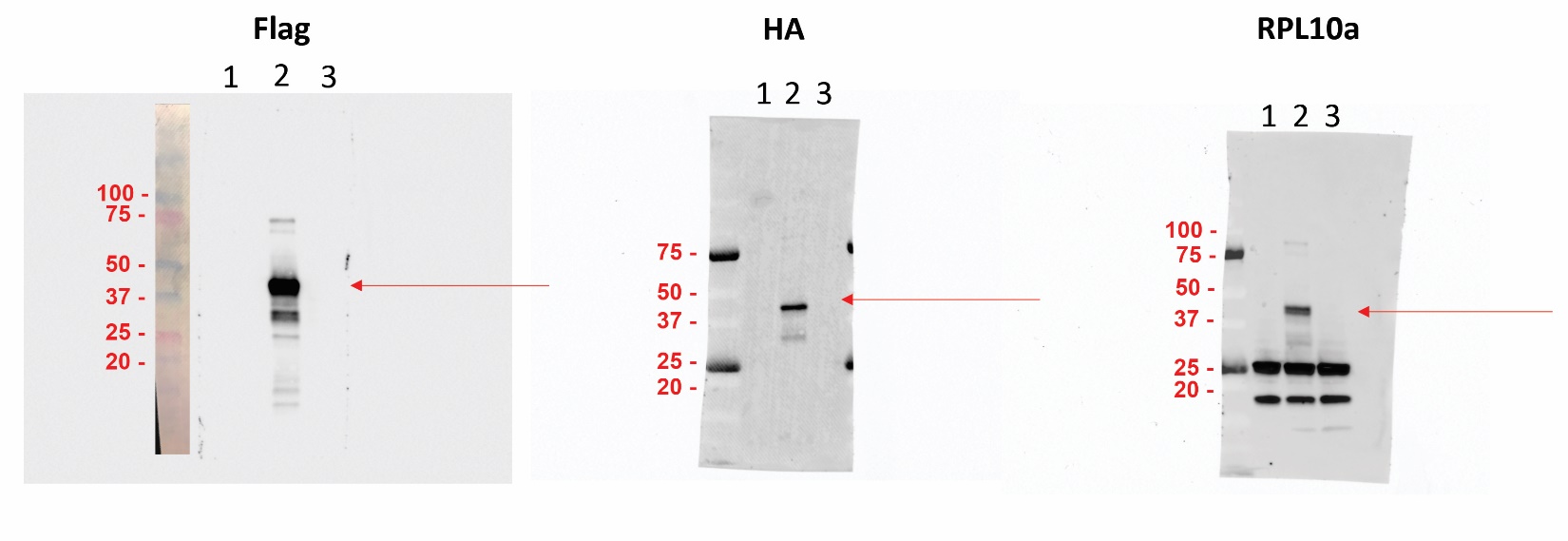
**

**Supplemental Figure 8**

**
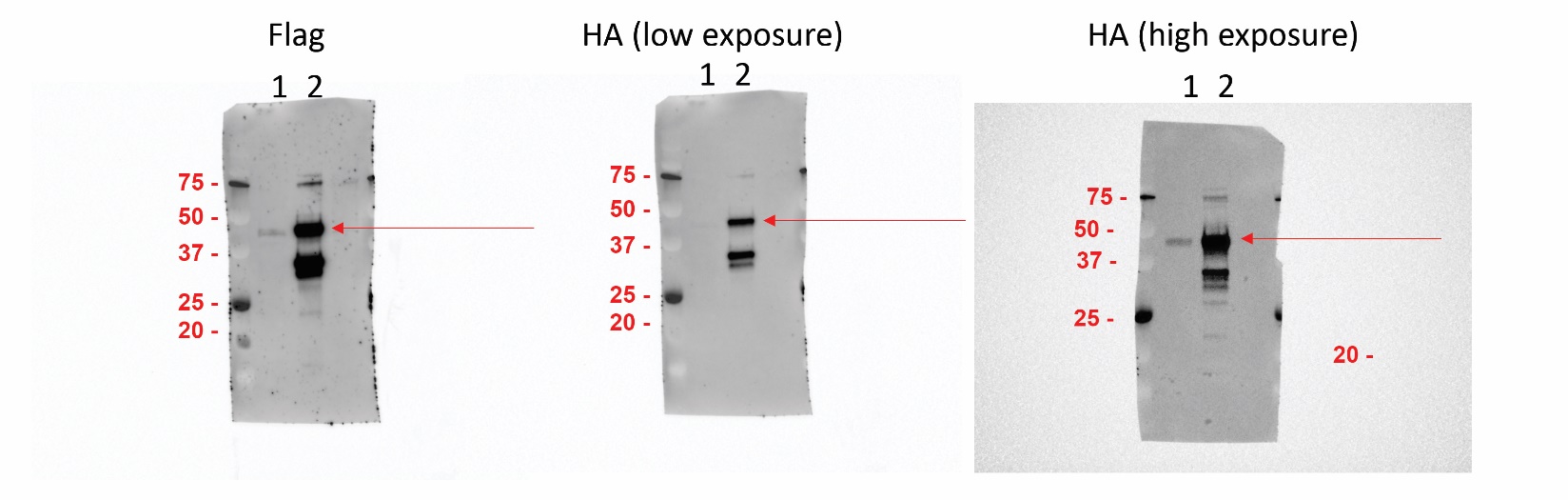
**

**Supplemental Figure 9**

**
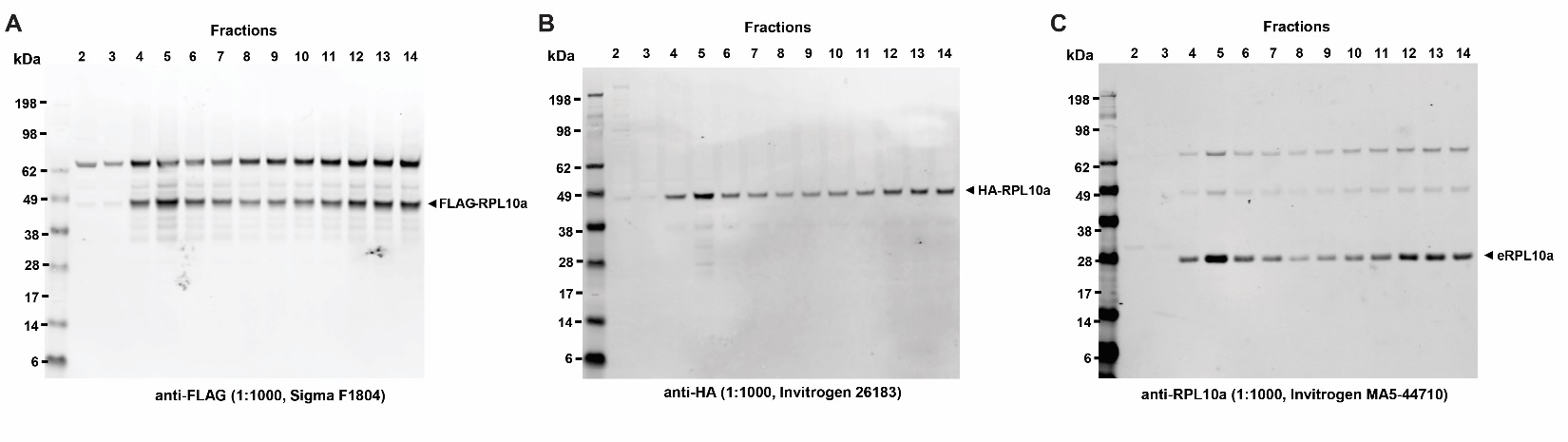
**

**Supplemental Figure 10**


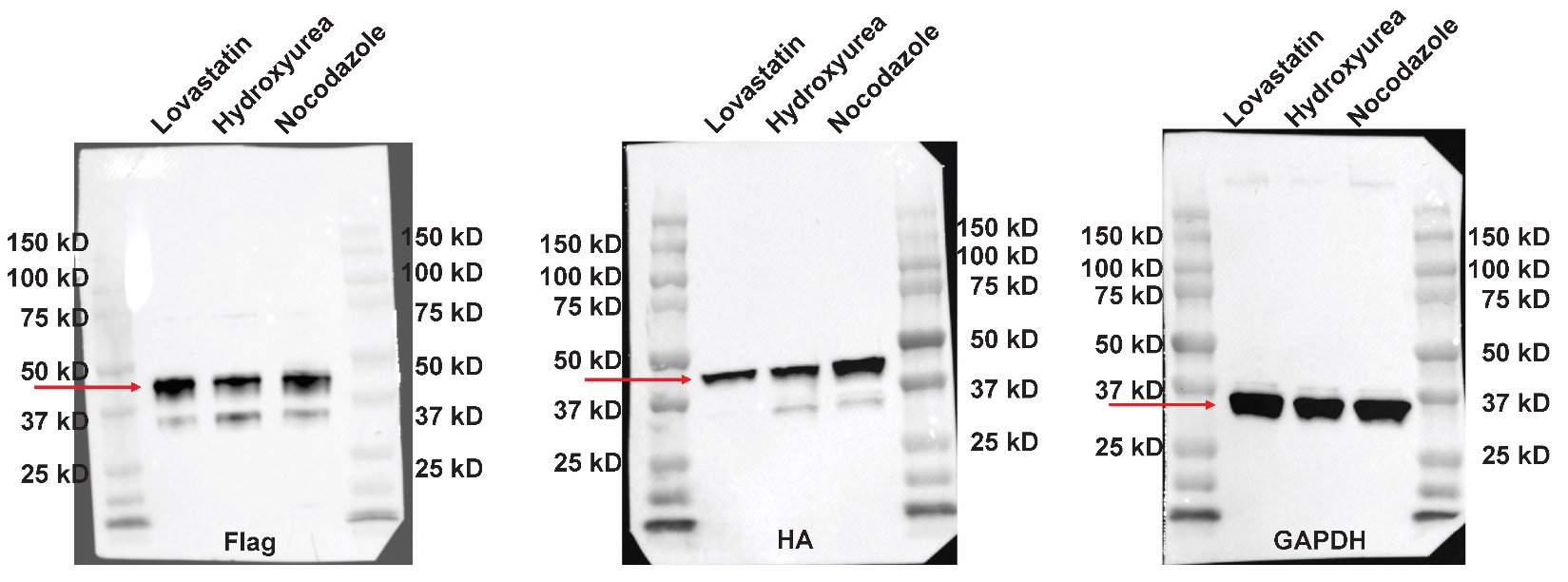


**Supplemental Figure 11**

**
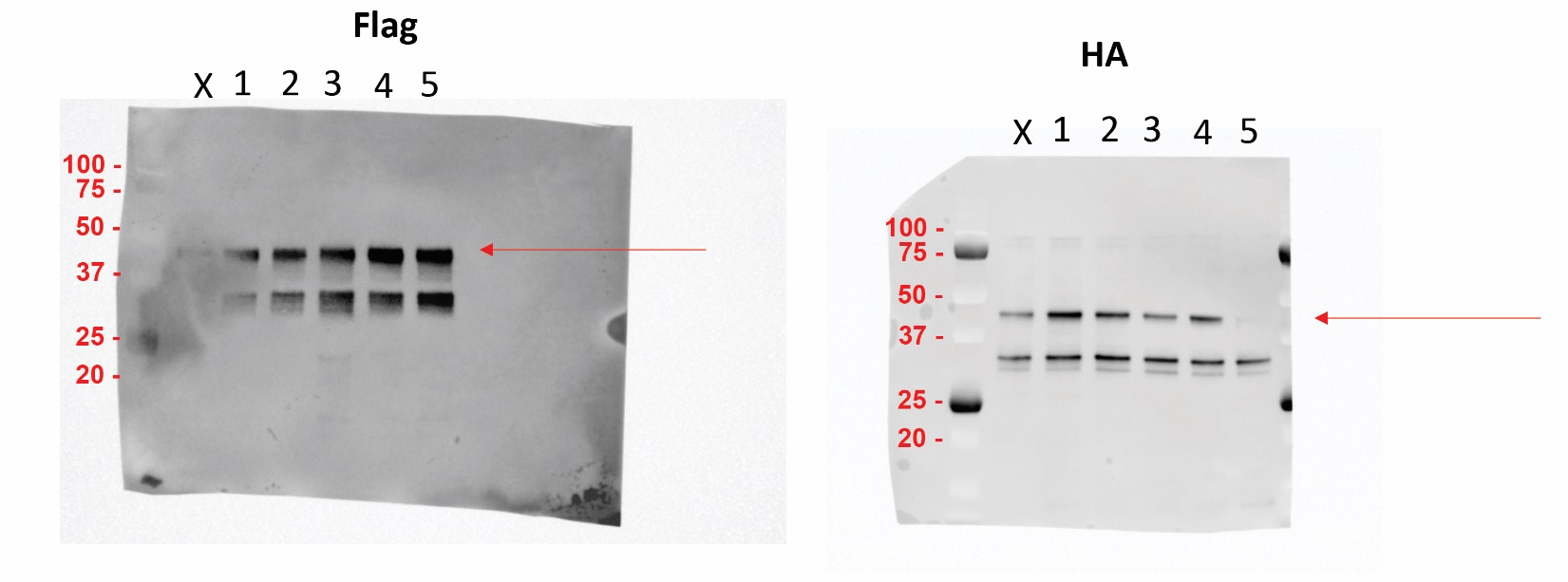
**

**Supplemental Figure 12**

**
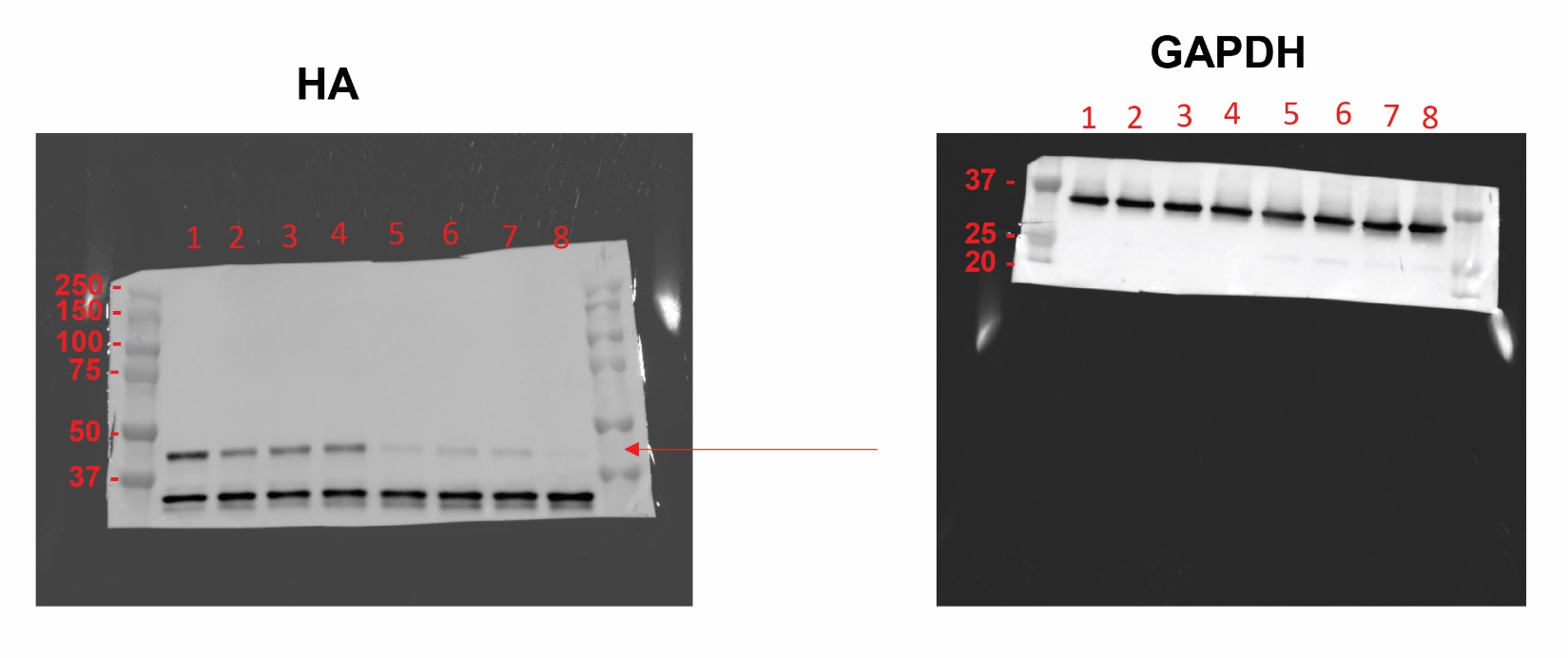
**
